# A DNA methylation signature in the stress driver gene *Fkbp5* indicates a neuropathic component in chronic pain

**DOI:** 10.1186/s13148-023-01569-8

**Published:** 2023-09-30

**Authors:** Maria Maiarù, Richard J. Acton, Eva L. Woźniak, Charles A. Mein, Christopher G. Bell, Sandrine M. Géranton

**Affiliations:** 1 Department of Cell and Developmental Biology, University College London, London, WC1E 6BT UK; 2 MRC Lifecourse Epidemiology Unit, University of Southampton, Southampton, UK; 3 Human Development and Health, Institute of Developmental Sciences, University of Southampton, Southampton, UK; 4 William Harvey Research Institute, Barts & The London Faculty of Medicine, Charterhouse Square, Queen Mary University of London, London, EC1M 6BQ UK; 5 Genome Centre, Faculty of Medicine and Dentistry, Queen Mary University of London, London, E1 2AT UK; 6 Present Address: Department of Pharmacology, School of Pharmacy, University of Reading, Reading, UK; 7 Present Address: Cologne Excellence Cluster for Cellular Stress Responses in Ageing-Associated Diseases (CECAD), University of Cologne, Cologne, Germany

**Keywords:** *FKBP5*, DNA methylation, Chronic pain, Preclinical models, Vulnerability

## Abstract

**Background:**

Epigenetic changes can bring insight into gene regulatory mechanisms associated with disease pathogenicity, including chronicity and increased vulnerability. To date, we are yet to identify genes sensitive to epigenetic regulation that contribute to the maintenance of chronic pain and with an epigenetic landscape indicative of the susceptibility to persistent pain. Such genes would provide a novel opportunity for better pain management, as their epigenetic profile could be targeted for the treatment of chronic pain or used as an indication of vulnerability for prevention strategies. Here, we investigated the epigenetic profile of the gene *Fkbp5* for this potential, using targeted bisulphite sequencing in rodent pre-clinical models of chronic and latent hypersensitive states.

**Results:**

The *Fkbp5* promoter DNA methylation (DNAm) signature in the CNS was significantly different between models of persistent pain, and there was a significant correlation between CNS and peripheral blood *Fkbp5* DNAm, indicating that further exploration of *Fkbp5* promoter DNAm as an indicator of chronic pain pathogenic origin is warranted. We also found that maternal separation, which promotes the persistency of inflammatory pain in adulthood, was accompanied by long-lasting reduction in *Fkbp5* DNAm, suggesting that *Fkbp5* DNAm profile may indicate the increased vulnerability to chronic pain in individuals exposed to trauma in early life.

**Conclusions:**

Overall, our data demonstrate that the *Fkbp5* promoter DNAm landscape brings novel insight into the differing pathogenic origins of chronic pain, may be able to stratify patients and predict the susceptibility to chronic pain.

**Supplementary Information:**

The online version contains supplementary material available at 10.1186/s13148-023-01569-8.

## Background

Chronic pain is a significant burden to society. In the UK alone, it affects 13–50% of adults, with at least 10% of those living with moderate to severe disabling pain [1, 2]. Importantly, chronic pain is not only limited to older age groups and affects up to 30% of those aged 18 to 39 years [2, 3]. Unfortunately, managing this condition remains a major challenge for clinical practice. Failure to diagnose and classify chronic pain patients may certainly contribute to the lack of efficacy of present therapies that are also often accompanied by significant side effects, including the potential for addiction [4, 5]. An opportunity for better pain management lies with strategies preventing the transition from acute to chronic pain. Unfortunately, identifying vulnerable individuals and preventing their pain to persist remains challenging [6, 7]. Thus, understanding more precisely the mechanisms involved in the pathogenesis of chronic pain may uncover novel therapeutic avenues as well as opportunities for more targeted personalised medicine.

The protein FKBP51, encoded by the gene *FKBP5*, is a regulator of the stress axis [8, 9]. Its genetic deletion and pharmacological blockade alleviate persistent pain states in rodents [10–12], while identified genetic variants have been associated with pain sensitivity after trauma in early small-scale human studies [13–15]. However, these genetic findings have not, to date, been replicated in larger biobank-scale genome-wide association studies. Importantly, persistent pain driven by FKBP51 is associated with a rapid decrease in *Fkbp5* DNA methylation (DNAm) in the rodent spinal cord which correlates with the spinal increase of the FKBP51 protein that can be locally prevented to relieve established persistent pain [10, 11]. Whether this change in DNAm is maintained together with the hypersensitive state has not been explored, but compelling evidence for a role of *FKBP5* DNAm dysregulation in human chronic diseases has recently been provided through genome-wide DNA studies [16–20].

Especially relevant in the context of preventive approaches for the management of chronic pain, early work from Klengel et al. indicated the potential that early life trauma in humans may lead to a decrease in *FKBP5* DNAm, an epigenetic change that primes *FKBP5* for hyper-responsiveness and may increase the susceptibility to post-traumatic stress disorder (PTSD) in adulthood [21]. We hypothesise that similar processes could underlie the vulnerability to chronic pain [22], but we do not know whether stress exposure could induce a change in DNAm at spinal cord level. Such a reduction in DNAm could prime *FKBP5* for hyper-responsiveness to injury in later life, therefore increasing the likelihood of developing persistent pain. This would provide a mechanistic basis for the well-described link between early life trauma and increased susceptibility to chronic pain [23].

The hypothesis of this project was that significant trauma of physical or emotional nature could induce a long-lasting change in the DNAm profile of the *FKBP5* gene in the central nervous system (CNS), which could maintain an individual in a hypersensitive state, or leave an individual susceptible to chronic pain. This was investigated through the use of pre-clinical models of persistent pain and of latent states of hypersensitivity.

## Results

Results are summarised in a graphical abstract (Additional file 1: Fig. S1).

### Ankle joint inflammation and nerve injury have different *Fkbp5* promoter DNAm landscape

**Fig. 1:**
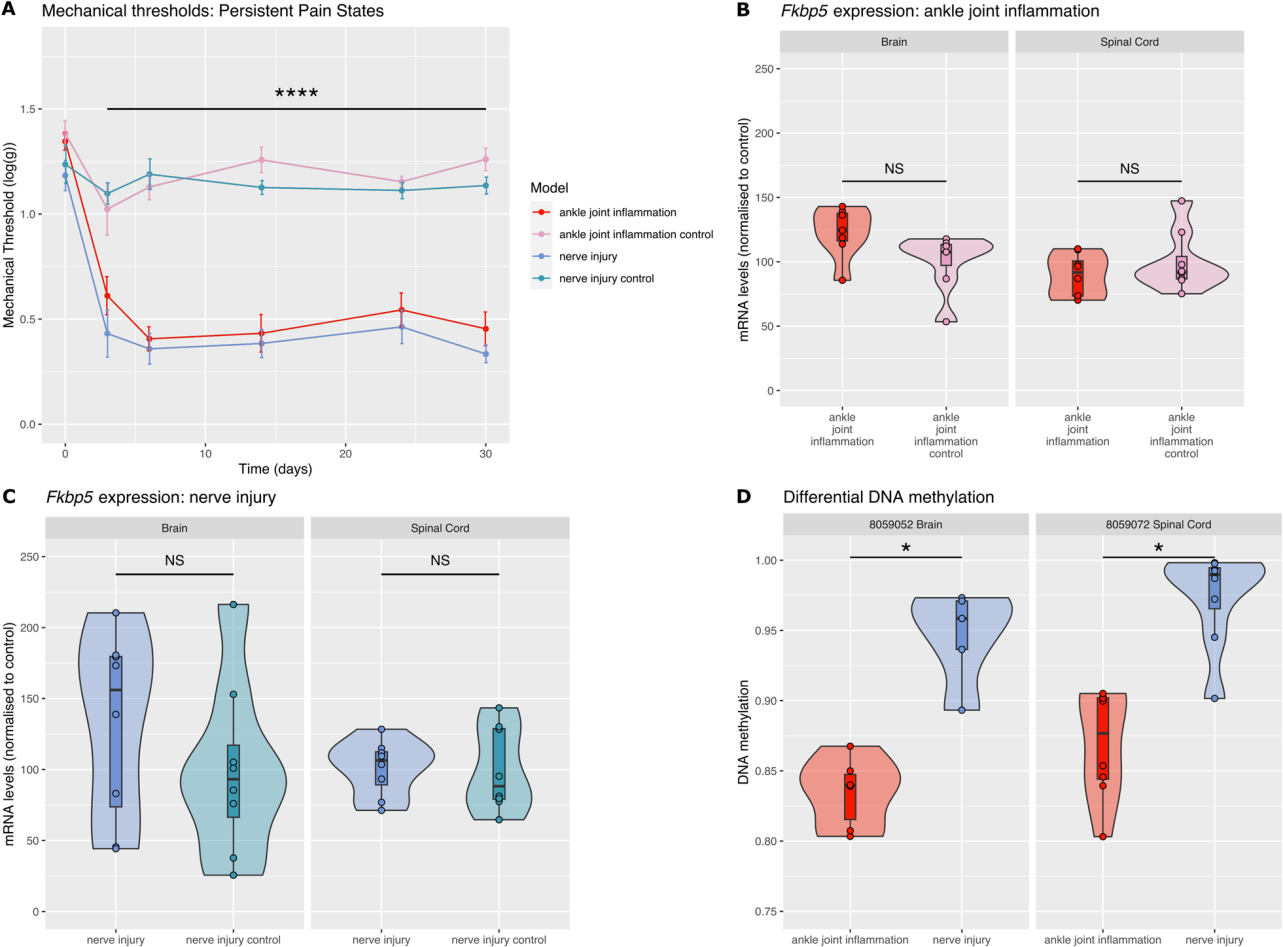
Ankle joint inflammation and nerve injury are associated with different *Fkbp5* promoter DNA methylation landscape. **A** Adult male rats were injected with an inflammatory agent in the ankle joint or had spared nerve injury surgery on day 0 (N = 8/8/8/8). Both models induced significant mechanical hypersensitivity compared with their control groups, but there was no difference in the mechanical hypersensitivity between the two models (****P < 0.0001, ankle joint inflammation *vs* ankle joint inflammation control and nerve injury *vs* nerve injury control). **B**, **C** There was no significant change in *Fkbp5* mRNA expression in the spinal cord and the hippocampus 30 days following inflammation and 30 days following nerve injury. mRNA levels were quantified by RTqPCR (N = 8/8). Data was normalised to control average. **D** We identified two CpG loci (cytosines at 8,059,052 & 8,059,072) with different levels of methylation between CFA and SNI tissue, in the brain and in the spinal cord, respectively (*P < 0.05)

Inflammation of the ankle joint and nerve injury induced long-lasting mechanical hypersensitivity in adult male rats (Fig. 1A; Day3 to Day30: ankle joint inflammation vs ankle joint inflammation control: F_1,15_ = 84, p < 0.0001; nerve injury vs nerve injury control: F_1,11_ = 48; p < 0.0001). There was no difference in the degree of mechanical hypersensitivity induced by the two models. We had previously found that both these models induced an upregulation of *Fkbp5* mRNA expression in the dorsal horn within few hours of the initiation of the pain states [10, 11]. Here, using RTqPCR to measure *Fkbp5* mRNA levels in the maintenance stage of the pain states, we found no change in expression of *Fkbp5* in the hippocampus or in the dorsal horn in both models when compared to their respective control groups (Fig. 1B,C).

Overall, of all targeted loci, reliable results passing QC were generated for 90 CpGs. Using the RnBeads analytic pipeline Wald test with an FDR correction and with correction for major neuronal cell-type proportions (see Methods), we identified CpGs with greater levels of methylation in the animals with nerve injury compared with animals with ankle joint inflammation (Fig. 1D): one CpG in the hippocampus at position chr20:8,059,052 (DNAmΔ = 11.2%; FDR-corrected p = 0.018, effect size 1.73 [S.E. 0.28]) and one CpG in the spinal cord at position chr20:8,059,072 (DNAmΔ = 10.5%; FDR-corrected p = 0.023, effect size 1.62 [S.E. 0.28]) (Fig. 1D). While there was no difference in DNAm between the animals with ankle joint inflammation and their control group at these sites, there was a trend for an increase between animals with nerve injury and their own controls at each CpG site (DNAmΔ = 3.2%; at positions chr20:8,059,052 (hippocampus) and DNAmΔ = 8.5% at chr20:8,059,072 (spinal cord), Additional file 1: Table S1) and a small change in the opposite direction between inflammation and controls (DNAmΔ = − 0.6%; at positions chr20:8,059,052 and DNAmΔ = − 1.9% at chr20:8,059,072, Additional file 1: Table S1), suggesting that the neuropathic animals were likely to have a signature different from all control animals and animals with an inflammatory pain state.

These two CpGs are located just beyond defined CpG shore regions (≤ 2 kb from CGI) within shelf regions (> 2 kb and ≤ 4 kb from the CGI, see Table 1). We then interrogated the DNA sequence that the two CpGs (chr20:8,059,052 and chr20:8,059,072) reside centrally within for potential Transcription Factor Binding Site (TFBS) motifs. Including ± 10 bp of sequence around the probed cytosines, we explored the Transfac 2010.1 Vertebrate database for potential TFBS motifs via the Transcription Factor Affinity Prediction (TRAP) v3.0.5 tool ([24], see Methods). This identified three potential TFBSs for the two CpGs (see Additional file 1: Table S2). This included motifs for the TFs (1) E2F1 (E2F transcription factor 1), which was found elevated in the blood of patients with spinal cord injury-induced neuropathic pain [25] and (2) AP1, recently identified as a key gene associated with neuropathic pain in the spinal cord [26].

**Table 1:** Location of significant DNA methylation difference between tissue from animals with ankle joint inflammation and nerve injury

Significant Cytosine (rn6)	Location	Distance to CGI (bp)	Nearest CGI	TFBS motifs (±10 bp) Transfac include	Mouse Syntenic Loci Regulatory Prediction (CNS)
chr20:8,059,052	Intronic CGI shelf	2,278	chr20:8,061,330–8,062,154	E2F1	Poised Enhancer/Quiescent Gene
chr20:8,059,072	Intronic CGI shelf	2,258	chr20:8,061,330–8,062,154	AP1	Poised Enhancer/Quiescent Gene

Includes (Left to Right): Location of Cytosine (rn6 build); Location annotation; Distance to nearest CpG Island (CGI); Co-ordinates of nearest CGI; Highlighted enriched Transcription Factor Binding Site motifs in ± 10 bp window centred on CpG via Transfac (TRAP analysis [24]; and observed Chromatin Segmentation signature in the syntenic Mouse location (GRCm38/mm10, via LiftOver) in developmental central neural tissue [27])

Next, we looked for epigenomic evidence of regulatory activity at these CpG loci. Unfortunately, no Rat-specific Chromatin Segmentation currently exists, so we lifted the ± 10 bp sequences over to mouse genome (via UCSC LiftOver) where Chromatin Segmentation has been performed in developing mouse tissue (Encode Phase III)[27]. This revealed that these two CpGs resided within potential poised enhancer as well as quiescent gene signatures in various developing neural tissues (Additional file 1: Fig. S2).

### DNAm levels at chr20:8,059,052 are correlated in the hippocampus, spinal cord and peripheral blood

**Fig. 2:**
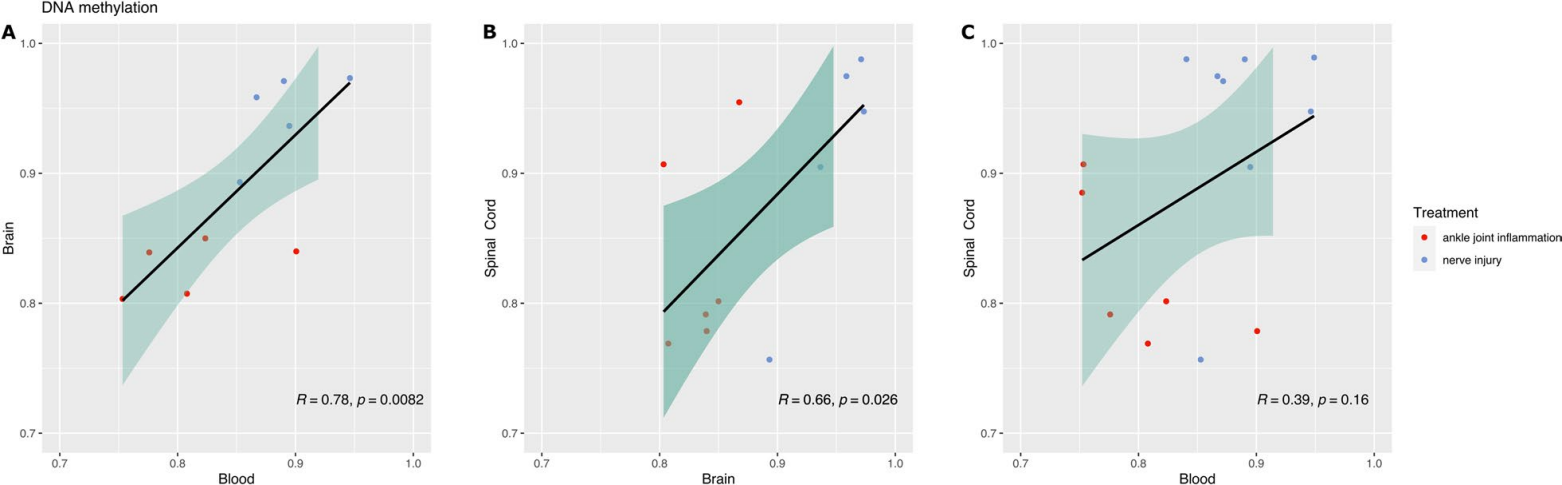
DNAm at chr20:8,059,052 in the hippocampus correlates with DNAm in the blood and in the spinal cord. **A**, **B** There is a significant correlation between DNAm in the brain and the blood: Pearson’s correlation P = 0.008, and between the brain and the spinal cord: Pearson’s correlation: P = 0.026. **C** The correlation between DNAm in the blood and the spinal cord did not reach significance

We observed that, while statistically not significant, the CpG at position chr20:8,059,052 was the second CpG—after chr20:8,059,072—most likely to show a change in DNAm in spinal cord tissue (DNAmΔ = 11.9%; FDR-corrected p = 0.109, effect size = 1.17 [S.E. = 0.41]). Furthermore, while we were not able to statistically adjust and explore potential cell-type-related DNAm variations in blood due to the lack of haematological cell-type data, we found an elevated level in DNAm at chr20:8,059,052 in the blood of neuropathic animals compared with the animals with inflammation of the same amplitude than that seen in the spinal cord and in the hippocampus (DNAmΔ = 8.5%; Bonferroni corrected T test p = 0.046, effect size = 1.37 [S.E. = 0.39]) (see Additional file 1: Table S1). Finally, there was a significant correlation between the degree of DNAm in the blood and the degree of methylation in the hippocampus, as well as between the degree of DNAm in the spinal cord and the degree of methylation in the hippocampus (Fig. 2, tissue comparison = brain *vs* blood, R = 0.78, p = 8.2 × 10^–3^; spinal cord *vs* brain, R = 0.66, p = 2.6 × 10^–2^; and spinal cord *vs* blood, R = 0.39, p = 0.16).

### Short-lasting inflammation in early life primes for hyper-responsiveness to inflammation in adulthood but does not lead to a change in *Fkbp5* mRNA expression or *Fkbp5* DNAm in adulthood

**Fig. 3:**
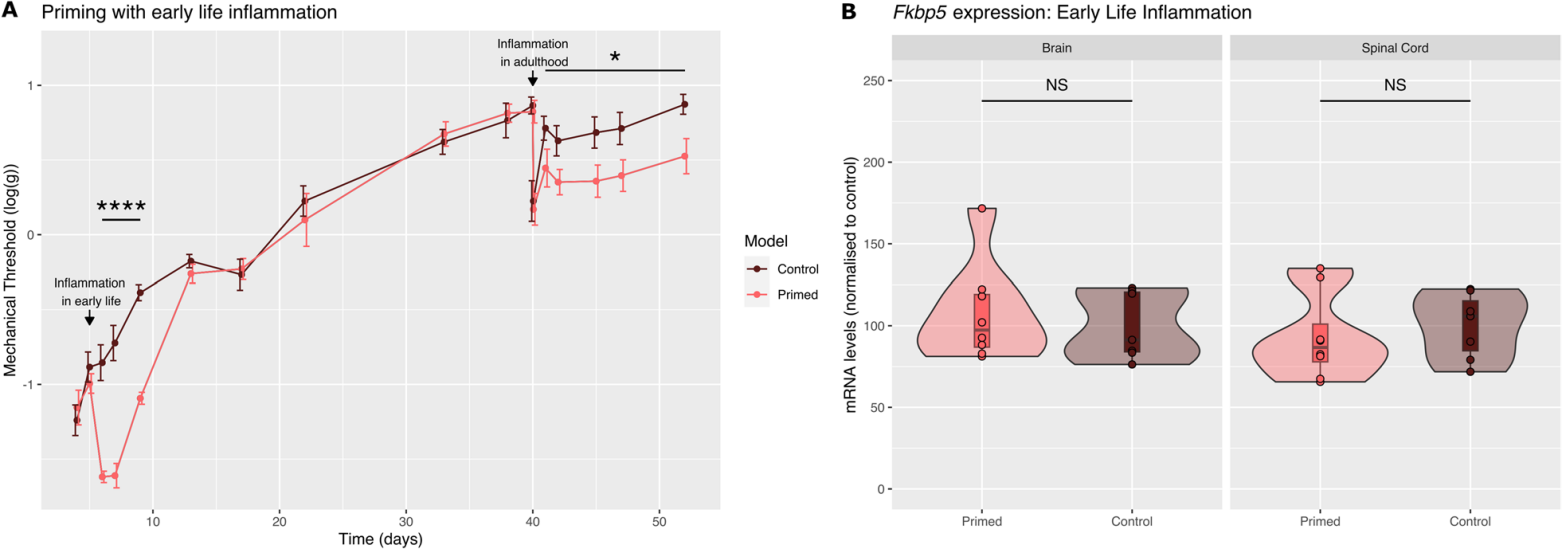
Intra-plantar inflammation in early life is not associated with long-term changes in *Fkbp5* mRNA expression. **A** Primed animals received a s.c. plantar injection of an inflammatory agent at postnatal day 5, and all animals received an injection of prostaglandin E2 at postnatal day 38. N = 8/8. Two-way ANOVA factor treatment, Day 5 to Day 9, F_1,14_ = 71.9, P < 0.0001; Day 41 to Day 52, F_1,14_ = 7.3, P < 0.05. Control vs primed. **B** There was no change in *Fkbp5* mRNA expression in the spinal cord or the brain 39 days after carrageenan injection (i.e. before prostaglandin E2 injection). mRNA levels were quantified by RTqPCR. N = 8/8. Data were normalised to control

Here, we used a model of early life inflammation in male rats at postnatal day 5. This induces short-lasting mechanical hypersensitivity and primes animals for hyper-responsiveness to injury in later life. This was indicated by the increased response to subsequent inflammation seen in adult primed animals compared with adult control animals (Fig. 3A). We then looked at *Fkbp5* mRNA expression and DNAm in control animals and primed animals before the induction of inflammation in adulthood, *i.e.* during the state of latent hyper-responsiveness. We found no change in *Fkbp5* gene expression (Fig. 3B) or *Fkbp5* DNAm with an FDR correction including an adjustment for cell-type proportions (data not shown).

### Early life adversity in the form of maternal separation primes for hyper-responsiveness to inflammation and leads to a decrease in *Fkbp5* mRNA expression and in *Fkbp5* DNAm in the spinal cord in adulthood

**Fig. 4:**
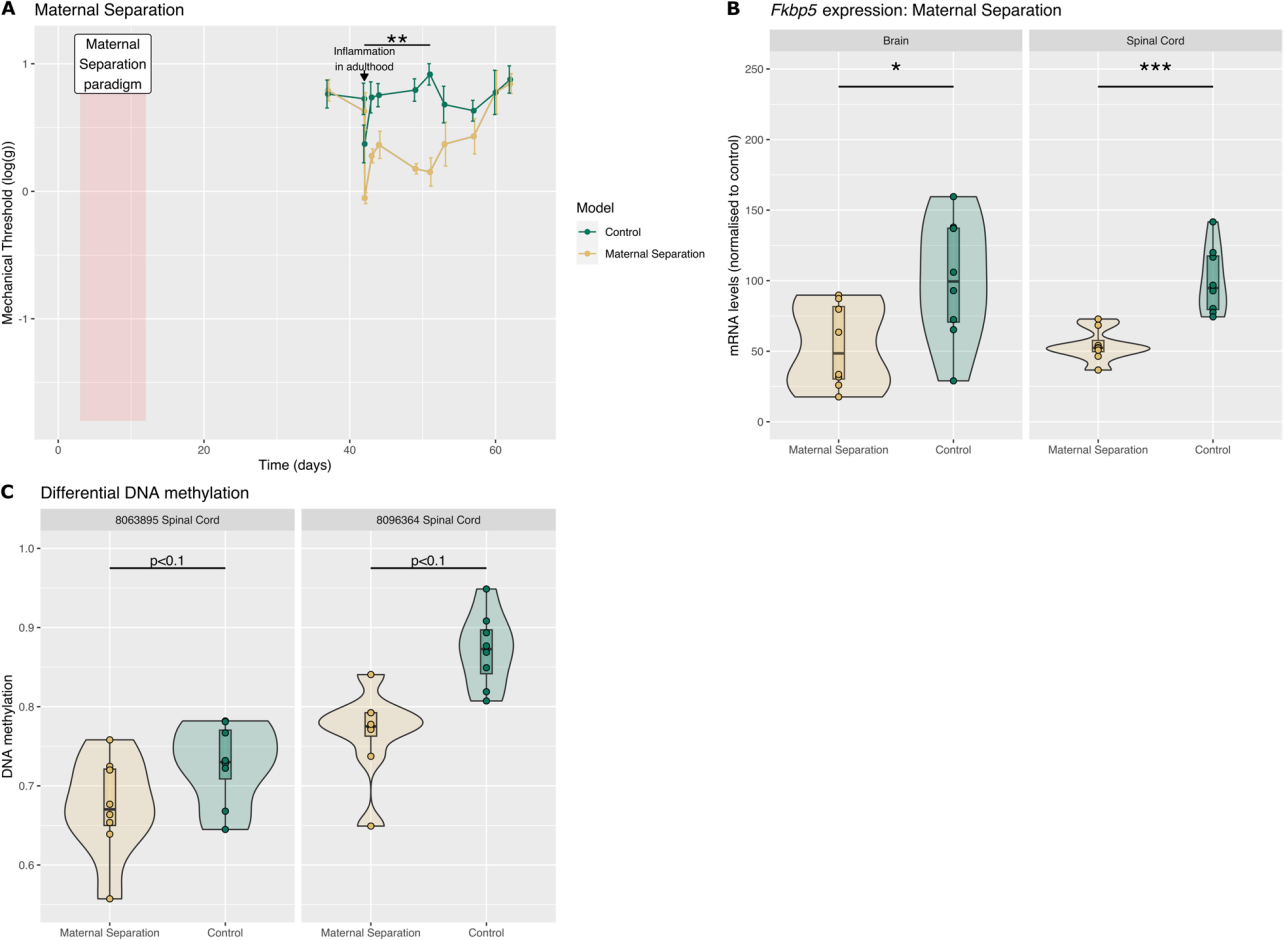
Maternal separation leads to a reduction in *Fkbp5* mRNA expression and *Fkbp5* DNAm. **A** Maternal separation from postnatal day 2 to 12 enhances mechanical hypersensitivity to inflammation in adulthood. All animals received a s.c. injection in the left hindpaw on Day 42 (also postnatal day 42). N = 8/8. Two-way ANOVA factor treatment, Day42 to Day49, F_1,14_ = 15.3, P < 0.01, control vs maternal separation. **B** There was a significant decrease in *Fkbp5* mRNA in both the brain and the spinal cord of rats with maternal separation at Day 40 (also postnatal day 40). mRNA levels were quantified by RTqPCR. N = 8/8. Data were normalised to control. Brain: control vs Mat Sep: P < 0.05 and spinal cord: control vs maternal separation: P < 0.001. **C** We identified two loci with different levels of DNAm between control and maternal separation with DNAm change > 1% and with FDR-corrected P trends < 0.1 (cytosines at chr20:8,063,895; chr20:8,096,364)

Finally, we explored the impact of early life adversity (maternal separation) on inflammation-induced hypersensitivity, *Fkbp5* mRNA expression and *Fkbp5* DNAm in adulthood. Maternal separation led to increased and prolonged mechanical sensitivity to inflammation in adulthood (Fig. 4A), as seen with early life inflammation. We then looked at *Fkbp5* mRNA expression and DNAm in control animals and animals that had experienced maternal separation only, *i.e.* before the induction of inflammation in adulthood. Unlike what we saw with the model of priming with inflammation (Fig. 3), maternal separation led to a long-lasting decrease in *Fkbp5* mRNA in both the hippocampus and the spinal cord (hippocampus: control vs maternal separation: P < 0.05 and spinal cord: control vs maternal separation: P < 0.001) (Fig. 4B). No CpG sites passed our strict FDR threshold including adjustment for cell-type proportions for statistically different DNAm levels between control and maternal separation tissue. However, we identified two CpG sites with DNAm change > 1% and with P trends < 0.1: CpG at chr20:8,063,895 (DNAmΔ = 5.4%; FDR-corrected p = 0.088, effect size = 0.88 [S.E. = 0.46]) and CpG at chr20:8,096,364 (DNAmΔ = 10.5%; FDR-corrected p = 0.071, effect size = 1.42 [S.E. = 0.35]) (Fig. 4C).

These CpGs also both reside within a CpG shore (Table 2), and these shore regions have been observed to both positively and negatively correlate with gene expression, potentially through interaction with certain activating or repressive transcription factors [28]. In our case, there was a reduction in DNAm with reduced gene expression.

**Table 2:** Location of major DNA methylation difference between control and maternal separation tissue

Significant Cytosine (rn6)	Location	Distance to CGI (bp)	Nearest CGI	TFBS motifs (±10 bp) Transfac include	Mouse Syntenic Loci Regulatory Prediction (CNS)
chr20:8,063,895	CGI shore	1,741	chr20:8,061,330–8,062,154	TFCP2	Quiescent/Polycomb Repressed/Poised Enhancer
chr20:8,096,364	CGI shore	935	chr20:8,097,299–8,097,607	EGR	Active Promoter/Polycomb Repressed (Bivalent)

Includes (Left to Right): Location of Cytosine (rn6 build); Location annotation; Distance to nearest CpG Island (CGI); Co-ordinates of nearest CGI; Highlighted enriched Transcription Factor Binding Site motifs in ± 10 bp window centred on CpG via Transfac (TRAP analysis); and observed Chromatin Segmentation signature in the syntenic Mouse location (GRCm38/mm10, via LiftOver) in developmental central neural tissue [27])

We next interrogated the DNA sequence for potential TFBSs as before and identified two potential motifs for the CpG at chr20:8,063,895 and eight potentials motifs for the CpG at chr20:8,096,364 (Additional file 1: Table S2). This included the motif for the early growth response (EGR) transcription family, and previously identified to be involved in nociceptive signalling [29, 30].

## Discussion

We have previously demonstrated that following noxious stimulation, *Fkbp5* is rapidly upregulated in the superficial dorsal horn, while *Fkbp5* DNAm is reduced [11]. We also previously showed that FKBP51 drives persistent pain of neuropathic and inflammatory origin and that its inhibition during the maintenance stage of these chronic conditions reduces mechanical hypersensitivity [10]. We report here that persistent pain of neuropathic and inflammatory origin induces long-lasting mechanical hypersensitivity of similar intensity but are not accompanied by a persistent upregulation of *Fkpb5*. Crucially, the DNAm landscape of *Fkbp5* was different between the two pain states, with data suggesting that *Fkbp5* promoter DNAm could indicate a neuropathic component to the chronic pain state. We also found that the latent state of hypersensitivity induced by inflammation (or physical trauma) in early postnatal age was not accompanied by a change in *Fkbp5* expression nor DNAm. However, there was a reduction of *Fkbp5* DNAm together with a decrease in *Fkbp5* expression following early life adversity (or emotional trauma), a paradigm that also primes for hyper-responsiveness to injury in adulthood. These results suggest that different mechanisms depending on the trauma may underlie the susceptibility to chronic pain.

Investigating *Fkbp5* promoter DNAm landscape after ankle joint inflammation and nerve injury revealed differences in DNAm between the two pain states. Interestingly, the difference in DNAm at chr20:8,059,052 between neuropathic animals and the animals with inflammation was significant in the hippocampus and also trended towards significance in the spinal cord. Indeed, the CpG at the position chr20:8,059,052 was the second CpG most likely to show a change in DNAm in spinal cord tissue. Moreover, our analysis showed a generalised difference of the same amplitude and direction at this CpG site in blood samples and there was a correlation between the DNAm level in the hippocampus and that in the blood and the spinal cord. These results suggested that changes in DNAm identified at this site may represent systemic changes occurring across multiple tissues.

*Fkbp5* DNAm was greater in neuropathic animals than in the animals with inflammation, and while there was no difference in DNAm between the inflamed animals and their controls, both at CpG at chr20:8,059,052 and at 8,059,072 in the brain and the spinal cord, there was a trend for an increase between neuropathic animals and their controls at these CpGs sites. The percentage methylation values for all controls and inflamed animals were also extremely similar, suggesting that DNAm was likely to have been increased by the nerve injury. While more work is required to confirm these early observations, the significant correlation between the DNAm levels in the CNS and the blood indicates that the DNAm profile of *Fkbp5* may have the potential to act as an indicator of a neuropathic component in chronic pain. Although our data suggest that there is no correlation between the DNAm level and the mechanical hypersensitivity associated with persistent pain, it may be that DNAm level at chr20:8,059,052 correlates with emotional behaviours such as anxiety and depression that often accompanie long-lasting pain states and differ across pain states.

Further investigation will be needed to reveal the functional relevance of an increase in DNAm at this site, but it is of interest to note that CpG chr20:8,059,052 may interact with the transcription factor E2F1. While the interaction between DNAm and transcription factors is complex, classically it is observed to inhibit TF binding, but can conversely also attract specific TFs [31]. E2F1 is particularly relevant in the context of neuropathic pain. It was found elevated in the blood of patients with spinal cord injury-induced neuropathic pain [25] and in the hippocampus of hypersensitive mice following spinal cord injury [32]. Moreover, its ablation reduced mechanical sensitivity in this context [33]. The increase in DNAm observed in our study would therefore potentially prevent upregulation of *Fkbp5* induced by E2F1 binding at the chr20:8,059,052 CpG after nerve injury. Indeed, *Fkbp5* mRNA was not found elevated in our CNS samples.

We also observed an increase in DNAm in the neuropathic compared with the inflammatory pain state in the spinal cord at chr20:8,059,072. This CpG resides within a binding motif for the TF AP1: Table S2), which also has great relevance to nociceptive signalling. Indeed, AP1 binding activity rapidly increases in the spinal cord after noxious stimulation and it has recently been identified as a key gene associated with neuropathic pain in the spinal cord [26]. Similarly to the CpG at chr20:8,059,052, an increase in DNAm at this cytosine could prevent upregulation of *Fkbp5* induced by AP1 and would explain the lack of *Fkbp5* upregulation observed in our samples.

Looking at the samples from animals that had been primed for hyper-responsiveness to inflammation in adulthood, we identified a decrease in DNAm at two CpGs after maternal separation, but not after physical trauma, even though both early life experiences had induced similar mechanical hypersensitivity in early life. It is of note that a recent small study in children with abusive or accidental injuries also reported divergence in buccal cell and peripheral blood DNAm levels, with lower levels in DNAm within the *FKBP5* promoter region when the injury was associated with abuse [34]. In our pre-clinical study, the changes in DNAm were observed at cytosines located at chr:8,063,895 and chr20:8,096,364. Interestingly, the later CpG is likely to interact with the EGR transcription factor family. The most studied member of this family in the context of nociceptive signalling is the immediate early gene Egr1 (Zif268), which is rapidly upregulated in the dorsal horn following noxious stimulation and, similarly to FKBP51, contributes to the maintenance of hypersensitive states [29, 30]. A decrease in DNAm at this CpG could suggest that increase binding of EGR1 may occur following subsequent noxious stimulation, which in turn may lead to greater levels of FKBP51 and greater hypersensitivity, as seen in maternally separated animals, following noxious stimulation. It is important to note that the decrease in DNAm at chr20:8,063,895 and chr20:8,096,364 was accompanied by a significant decrease in *Fkbp5* mRNA levels at baseline. However, the impact of this decrease in DNAm on *Fkbp5* mRNA expression following noxious stimulation has not been explored here. Overall, we found no correlation between the interrogated CpGs’ DNAm and expression levels of *Fkbp5* mRNA, suggesting a complex regulation of gene expression for the gene *Fkbp5*.

While we have identified a number of CpG loci indicative of potential differences in the pathogenic origin of chronic pain, the conclusions that can be drawn from this study may be limited. Firstly, while our findings from previous studies on the role of FKBP51 in the maintenance of chronic pain have so far suggested no sex differences, our findings on DNAm in male rats would need to be confirmed in females. Secondly, while this study provided very high-depth coverage of *Fkbp5* promoter region to follow on from our promising earlier results [10], other functional elements of the gene may also have relevance in the biological mechanisms studied here. Indeed, significant DNAm findings have been identified in a recent similar targeted DNAm analysis of *Fkbp5* in a murine model of early life stress exposure in both the frontal cortex and blood [35]. Furthermore, that study interrogated additional potential regulatory elements in this gene, including intronic and CTCF loci. Considering that changes in *FKBP5* DNAm have been observed with other mental health conditions [19, 36], it is likely that a more complete picture of the DNAm landscape, including that of other pain-related genes, may be necessary for a more precise understanding of chronic pain. Indeed, our results show that *Fkbp5* DNAm landscape might be more likely to correlate with the affective than with the sensory experience of chronic pain.

## Conclusion

While our previous work had highlighted FKBP51 as a new target for the treatment of chronic pain, this study is the first to propose *FKBP5* promoter DNAm landscape as a potential pathological indicator of neuropathic pain and of the susceptibility to chronic pain. These findings provide a unique opportunity to design and develop novel approaches for the management and prevention of chronic pain, a disabling condition with significant unmet clinical needs. CRISPR-mediated epigenome editing is now at the forefront of medical research [37] with in vivo single-gene approaches being pursued for the treatment of chronic pain [38], and we propose *FKBP5* as a suitable target for similar interventions.

## Methods

This study investigated the epigenetic profile of the gene *Fkbp5* using targeted bisulphite sequencing in rodent pre-clinical models of chronic and latent hypersensitive states.

### Animals

Subjects in all experiments were male Sprague–Dawley rats obtained from the colony at UCL. All rats were kept in their home cage in a temperature-controlled (20° ± 1° C) environment, with a light–dark cycle of 12 h. Home cages were enriched with tubes, nesting materials and chewing sticks. Food and water were provided ad libitum. All behavioural experiments were performed by the same experienced female experimenter, who was always blind to treatment group for all behavioural tests. Animals were allocated to a treatment group using a random number generator. Mechanical hypersensitivity was measured using a series of calibrated Von Frey’s monofilaments [11]. The threshold was determined by using the up–down method [39]. The data are expressed as log of mean of the 50% pain threshold ± SEM.

### Animal models

#### Ankle joint inflammation

We induced joint inflammation with the injection of Complete Freund’s Adjuvant (10 µl) in the ankle joint in adult male rats (250 g, 7-week old) as before [40]. Control (sham) animals were only put under anaesthesia for few minutes. For molecular analysis, tissues were collected 30 days after the injection. N = 8 per group.

####  Spared nerve injury

For the neuropathic pain model, we used the spared nerve injury model in adults male rats (250 g, 7-week old) as before [11, 41]. Control (sham) animals were put under anaesthesia, and the nerve was exposed but untouched before closing the wound. For molecular analysis, tissues were collected 30 days after the spared nerve injury surgery. N = 8 per group.

#### Physical priming with inflammation*Physical priming with inflammation*

The mechanical hypersensitivity that develops after plantar subcutaneous (s.c.) injection of carrageenan resolves within few hours in neonates at postnatal day 5. However, when these animals are re-injured later on in life, they display a greater mechanical hypersensitivity when compared with animals that have not previously been injected with carrageenan [42]. For priming, male rats received carrageenan (1ul/g of body weight) at postnatal day 5 in the left hindpaw and control animals were only put under anaesthesia for few minutes. All male rats received a plantar s.c. injection of prostaglandin E2 in the left hindpaw (100 ng/25ul) at postnatal day 40. For molecular analysis, tissues were collected at postnatal day 40, with no injection of prostaglandin E2. N = 8 per group.

#### *Emotional priming with early life adversity: maternal separation*

Stress in early life was induced in male rats by maternal separation, 3 h/day from postnatal day 2 to day 12 [43]. Control male rats stayed with the dam in their home cage. All experimental male rats received a plantar s.c. injection of prostaglandin E2 (100 ng/25ul) at postnatal day 42. For molecular analysis, tissues were collected at P40, with no prostaglandin E2 injection. N = 8 per group.

### DNA & RNA extraction

Following behavioural assessment, animals were terminally anaesthetised with CO_2_. CNS samples (full hippocampus and ipsilateral quadrant of the L4-L6 spinal cord segment) and blood were harvested from animals, and DNA and RNA were extracted from these same samples. We used the All Prep DNA/RNA/miRNA Universal Kit from Qiagen, https://www.qiagen.com/us/products/discovery-and-translational-research/dna-rna-purification/multianalyte-and-virus/allprep-dnarnamirna-universal-kit/?catno=80224 as per the manufacturer’s instructions.

### RTqPCR

Reactions were performed at least in triplicate, and the specificity of the products was determined by melting curve analysis. The ratio of the relative expression of target genes to the housekeeping gene Hgprt was calculated by using the 2^ΔCt^ formula as before [11].

### DNA methylation targeted bisulphite sequencing

Primers for targeted DNA methylation (DNAm) bisulphite sequencing were designed with MethPrimer 1.0 [44] to survey of the status of the potentially functional CpG dense promoter region of the *Fkbp5* gene. This includes the 3 upstream CpG islands (CGIs) and their shore regions (Additional file 1: Fig. S3). Parameters employed for the primer design were product size (minimum 125 bp; optimal 150 bp; maximum 175 bp), Primer Tm (min. 50°; opt. 55°; max. 60°), primer size (min 20 bp; opt. 25 bp; max 30 bp), and including at least 4 product CpGs. Twenty-one loci were initially targeted covering 90 CpGs (Additional file 1: Table S3).

The targeted sequencing loci were assessed for DNAm changes between the various animal models. Of all the targeted loci, reliable results passing QC were generated for 90 CpGs. Quality control included visualisation assessment pre- and post-read trimming via fastqc v0.11.5 (Andrews, S. FastQC, Babraham Bioinformatics, Cambridge, 2010) and multiqc v1.0[45]. Cutadapt v1.13 [46] and a custom perl5 script were employed to trim targeting primers, with then quality trimming via trim_galore 0.5.0 [47]. Bismark v0.20.0 [48], incorporating the bowtie2 v.3.1 [49] aligner, was used for DNAm calling. Alignment was performed against targeted loci ± 100 bp in the rat rn6 genome with a minimum coverage threshold of 25 reads. Differential methylation analysis was performed using RnBeads v2.0.1 [50].

### Cell-type heterogeneity correction and reported differentially methylated cytosines

To account for influence of the cell-type heterogeneity of the DNA samples, RTqPCR data were used to correct CNS samples for cell-type proportions. One correction was applied using as covariates Aif1 for glia, Aldhl1 for astrocytes and Olig1 for oligodendrocytes (Cell-Type Correction 1, CTC1). To check the robustness of our approach, another correction was also applied using as covariates CxCr1 for microglia, Aldhl1 for astrocytes and Olig1 for oligodendrocytes (Cell-Type Correction 2, or CTC2). CpGs for differentially methylated loci were tested with the Wald test as implemented in RnBeads [50].

Our final high-quality CpG results reported here were required to pass a threshold of (1) coverage greater than 300 reads for all samples within a group; (2) methylation difference greater than 1% and (3) P < 0.05 for both CTC1 and CTC2 (or P < 0.1 for the reported trend results). In the Results section, the P values for CTC1 are stated. We were unable to correct the blood samples for cell-type variation due to a lack of haematological data; however, these were considered as pilot data to explore the potential biomarker utility of blood cell-derived DNAm.

### Transcription factor binding motif enrichment

The Transcription Factor Affinity Prediction (TRAP) v3.0.5 [24] ‘single-sequence’ option was used to explore any enrichment for transcription factor (TF) binding motifs surrounding significant CpGs. The sequences around each CpG were expanded to ± 10 bp and the FASTA sequence extracted (rn6) via UCSC. The Transfac 2010.1 Vertebrate matrix set was interrogated with mouse_promoter set as background model.

### Supplementary Information

**Additional file 1:** Graphical abstract and supplementary data.

## Data Availability

Data are available for sharing on reasonable request. Contact the corresponding author.

## Abbreviations

DNAm: DNA methylation
CTC: Cell-type correction
TFBS: Transcription Factor Binding Site
