# Supplementary material for: A DNA methylation signature in the stress driver gene Fkbp5 indicates a neuropathic component in chronic pain
Source: Clin Epigenetics. 2023 Sep 30;15:155. doi: 10.1186/s13148-023-01569-8 (PMC10543848; doi:10.1186/s13148-023-01569-8)
Supplement: Supplementary file 1 — Additional file 1: Graphical abstract and supplementary data. [file 13148_2023_1569_MOESM1_ESM.docx]

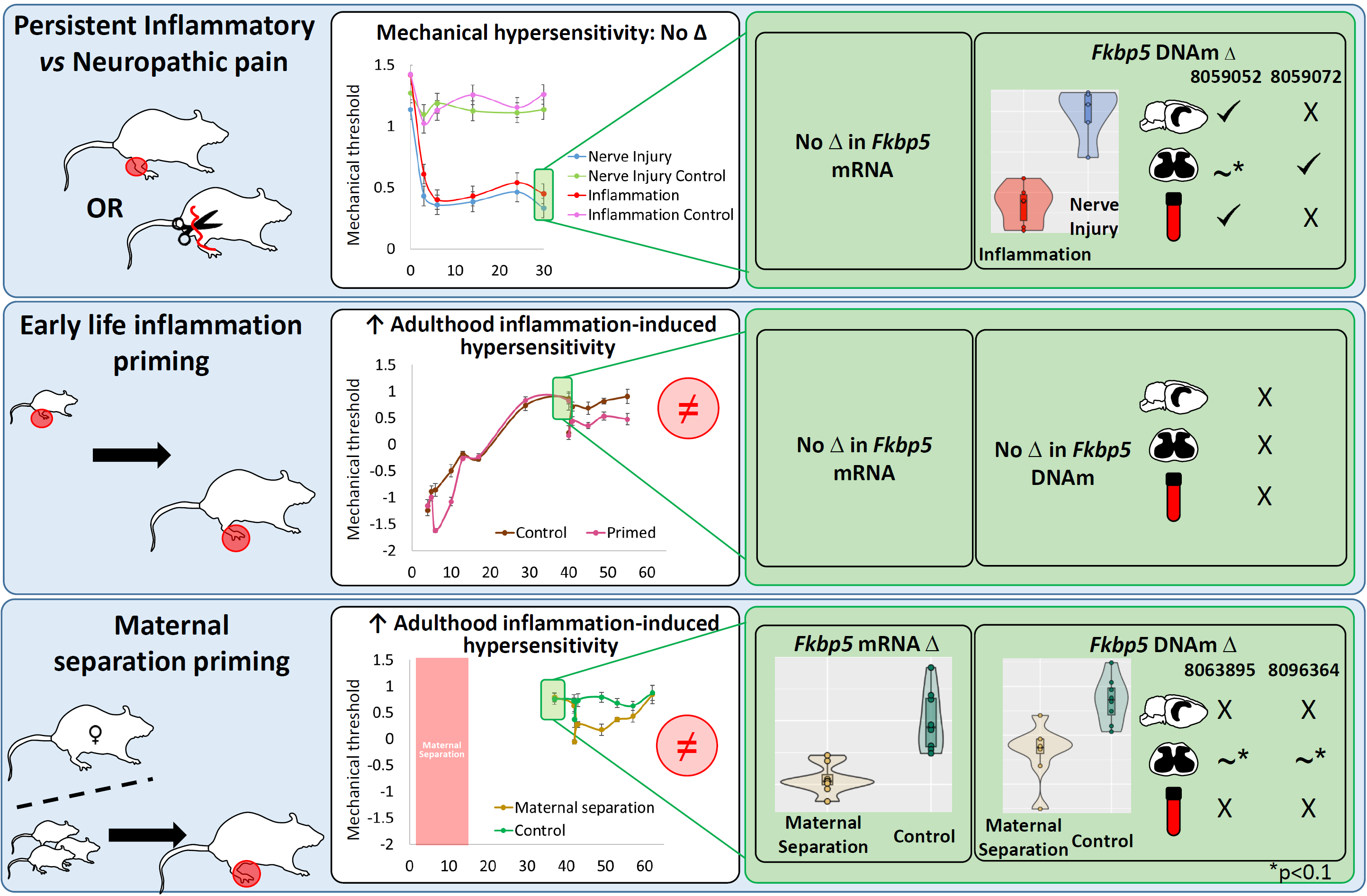


**Figure S1: Graphical abstract.** Overview of findings: *Fkbp5* promoter DNA methylation (DNAm) signature was significantly different between animals with inflammation and nerve injury. There was no change in *Fkbp5* mRNA and DNAm after early life inflammation. However, we found that maternal separation, which also promotes the persistency of inflammatory pain in adulthood, was accompanied by long-lasting reduction in *Fkbp5* mRNA and DNAm, suggesting that *Fkbp5* DNAm profile may indicate the increased vulnerability to chronic pain in individuals exposed to trauma in early life.

Chr20:8059052 Chr20:8059072


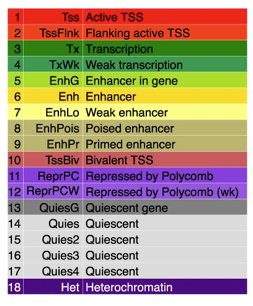


Figure S2. Chromatin Segmentation in Syntenic Mouse Regions. Top to Bottom: Sequence location in Rat (rn6); Sequence location in Mouse (GRCm38/mm10); Gencode Transcript Information (VM23); mouse CREs; CpG islands; Chromatin Segmentation in developing Forebrain, Midbrain, Hindbrain, and Neural Tube. Legend colour code for 18-state Segmentation.

Figure S3. Location of sequencing probes of 3 5’CpG Island regions and plausible promoter regions of *Fkbp5* gene displayed in UCSC Browser. Layers from top: Scale in Rat (rn6) build; Genomic location chr20:7,969,338-8,108,322; Location of Targeted Sequencing regions for DNAm analysis (purple); Location of CpG Islands (green); GC Percentage indicating increase at CpG Island locations; Location of Fkbp5 from UCSC, Rat Spliced and unspliced ESTs; Mouse mm39 Gencode (vm28) LiftOver; Conservation across 20 Vertebrates (PhyloP) and as individual Multiz Alignments.

Table S1. DNA methylation levels in injured and control animals at CpG sites chr20: 8,059,052 and chr20: 8,059,072. % DNAm average ± SEM.

| **CpG/tissue** | **Inflammation** | **Inflammation**  **control** | **Nerve Injury** | **Nerve injury control** | **Nerve Injury Δ Inflammation** | **Nerve Injury Δ**  **Control** | **Inflammation Δ**  **Control** |
| --- | --- | --- | --- | --- | --- | --- | --- |
| **8,059,072/spinal cord** | 86.9 ± 1.3 % | 88.8 ± 2.2 % | 97.3 ± 1.1 % | 88.8 ± 4.2 % | 10.5 % | 8.5% | -1.9% |
| **8,059,052/spinal cord** | 82.1 ± 2.9 % | 85.6 ± 2.6 % | 94.0 ± 2.6 % | 86.6 ± 5.2 % | 11.9 % | 7.4% | -3.5% |
| **8,059,052/hippocampus** | 83.5 ± 0.9 % | 84.0 ± 1.4 % | 94.6 ± 1.3 % | 91.5 ± 5.3 % | 11.2% | 3.2% | -0.6% |
| **8,059,052/blood** | 80.4 ± 1.7 % | 81.6 ± 2.0 % | 88.9 ± 1.3 % | 83.1 ± 2.0 % | 8.5% | 5.8% | -1.2% |

**Table S2.** **Transfac Motif prediction via TRAP.**

| **CpG** | **Rank** | **P-value** | **Matrix ID** | **Matrix name** |
| --- | --- | --- | --- | --- |
| 8,059,052 | 1 | 0.0218 | M00428 | V$E2F1_Q3 |
| 8,059,052 | 2 | 0.0409 | M01598 | V$ZBED6_01 |
| 8,059,052 | 3 | 0.0448 | M00431 | V$E2F1_Q6 |
| 8,059,072 | 1 | 0.0189 | M00495 | V$BACH1_01 |
| 8,059,072 | 2 | 0.0309 | M00174 | V$AP1_Q6 |
| 8,059,072 | 3 | 0.0496 | M00199 | V$AP1_C |
| 8,063,895 | 1 | 0.0128 | M00720 | V$CACBINDINGPROTEIN_Q6 |
| 8,063,895 | 2 | 0.0344 | M00072 | V$CP2_01 |
| 8,096,364 | 1 | 0.0171 | M00776 | V$SREBP_Q3 |
| 8,096,364 | 2 | 0.0188 | M00497 | V$STAT3_02 |
| 8,096,364 | 3 | 0.023 | M00690 | V$AP3_Q6 |
| 8,096,364 | 4 | 0.0249 | M00494 | V$STAT6_01 |
| 8,096,364 | 5 | 0.0258 | M00979 | V$PAX6_Q2 |
| 8,096,364 | 6 | 0.0267 | M00807 | V$EGR_Q6 |
| 8,096,364 | 7 | 0.0283 | M00246 | V$EGR2_01 |
| 8,096,364 | 8 | 0.0496 | M00378 | V$PAX4_03 |

Table S3. Investigated CpG regions (Rat assembly rn6)

| chromosome | start | stop | CpGs covered |
| --- | --- | --- | --- |
| chr20 | 8059015 | 8059131 | 4 |
| chr20 | 8062075 | 8062179 | 9 |
| chr20 | 8062233 | 8062336 | 3 |
| chr20 | 8063885 | 8063995 | 5 |
| chr20 | 8096103 | 8096195 | 2 |
| chr20 | 8096316 | 8096391 | 2 |
| chr20 | 8096800 | 8096880 | 5 |
| chr20 | 8096916 | 8096993 | 5 |
| chr20 | 8097096 | 8097190 | 2 |
| chr20 | 8097291 | 8097389 | 12 |
| chr20 | 8097867 | 8097945 | 2 |
| chr20 | 8098183 | 8098283 | 3 |
| chr20 | 8099134 | 8099255 | 3 |
| chr20 | 8100968 | 8101069 | 2 |
| chr20 | 8101327 | 8101429 | 2 |
| chr20 | 8101818 | 8101915 | 2 |
| chr20 | 8102706 | 8102783 | 4 |
| chr20 | 8102783 | 8102870 | 8 |
| chr20 | 8103416 | 8103525 | 5 |
| chr20 | 8103670 | 8103774 | 7 |
| chr20 | 8104402 | 8104523 | 3 |

Additional file 1 caption:

Graphical abstract and supplementary data.
